# Prostate volume changes during permanent seed brachytherapy: an analysis of intra-operative variations, predictive factors and clinical implication

**DOI:** 10.1186/1748-717X-8-177

**Published:** 2013-07-09

**Authors:** Ciprian Chira, Guila Delouya, Sandra Larrivée, Jean-Francois Carrier, Daniel Taussky

**Affiliations:** 1 Departement of Radiation Oncology, Centre hospitalier de l’Université de Montréal (CHUM), Notre-Dame Hospital, 1560 Sherbrooke St. E., Montreal, QB H2L 4M1, Canada; 2 CRCHUM-Centre de recherché du Centre Hospitalier de l’Université de Montréal, Montréal, Canada

**Keywords:** Prostate, Permanent seed brachytherapy, Intra-operative edema

## Abstract

**Background:**

To determine prostate volume (Pvol) changes at 3 different time points during the course of I^125^ permanent seed brachytherapy (PB). To assess the impact of these changes on acute urinary retention (AUR) and dosimetric outcome.

**Methods:**

We analyzed 149 hormone-naïve patients. Measurements of the prostate volume were done using three-dimensional transrectal ultrasound (3D-TRUS) in the operating room before insertion of any needle (V1), after the insertion of 2 fixation needles with a harpoon (V2) and upon completion of the implant (V3). The quality of the implant was analyzed with the D90 (minimum dose in Grays received by 90% of the prostate volume) at day 30.

**Results:**

Mean baseline prostate volume (V1) was 37.4 ± 9.6 cc. A volume increase of >5% was seen in 51% between V1-V2 (mean = 2.5 cc, p < 0.01), in 42% between V2-V3 (mean = 1.9 cc, p < 0.01) and in 71% between V1-V3 (mean = 4.5 cc, p < 0.01). Pvol changes caused by insertion of the fixation needles were not statistically different than those caused by the implant itself (p = 0.23).

In multivariate linear regression analysis, baseline Pvol is predictive of Pvol changes between V2 and V1 and V3 and V1 but not between V3 and V2. The extent of prostate swelling had an influence on D90. An increase of 10% in prostate volume between V1 and V2 results in an increase of D90 at Day 30 by 11.7%. Baseline Pvol (V1) was the only predictor of the duration of urinary retention in both univariate and multivariate (p = 0.04) regression analysis.

**Conclusions:**

A large part of intraoperative swelling occurs already after the insertion of the fixation needles. This early prostate swelling predicts for D90 but not for AUR.

## Background

Prostate swelling associated with I^125^ permanent seed brachytherapy (PB) is considered a risk factor for suboptimal post implant dosimetry [1], decrease probability of tumor control [2] and higher toxicity rates [3]. The time course of this phenomenon has been previously described. But different imaging modalities (CT, MRI, TRUS) were used and compared to each other and some patients received perioperative medication that could influence prostate swelling [1,4-6]. None of these studies analyzed the clinical impact of prostate swelling.

The authors believe that knowing the intra-operative extent of Pvol changes, its predictive factors, and the post-operative impact of Pvol changes could help identify future patients who might need adjustments in planning to correct for prostate swelling. The present study is the first analysis using 3 consecutive volume measurements with TRUS to investigate risk factors for prostate swelling as well as its clinical implications.

## Methods

### Study patients

A total of 149 consecutive patients with low- or intermediate risk prostate carcinoma (≤T2b, Gleason score ≤ 7 and prostate-specific antigen ≤18) underwent prostate PB as monotherapy using an intraoperative planning (IO) approach. No patient had received any antiandrogen therapy before implantation. Patients were treated between January 2006 and February 2009 with 125-Iodine loose seed implants, with a typical activity between 0.4-0.65 mCi, without the addition of external beam radiation therapy. The prescribed dose was 144 Gy in all cases. The study received institutional review board approval of our hospital, Notre-Dame Hospital, Montreal (CER 12.092).

### Brachytherapy technique

**Figure 1:**
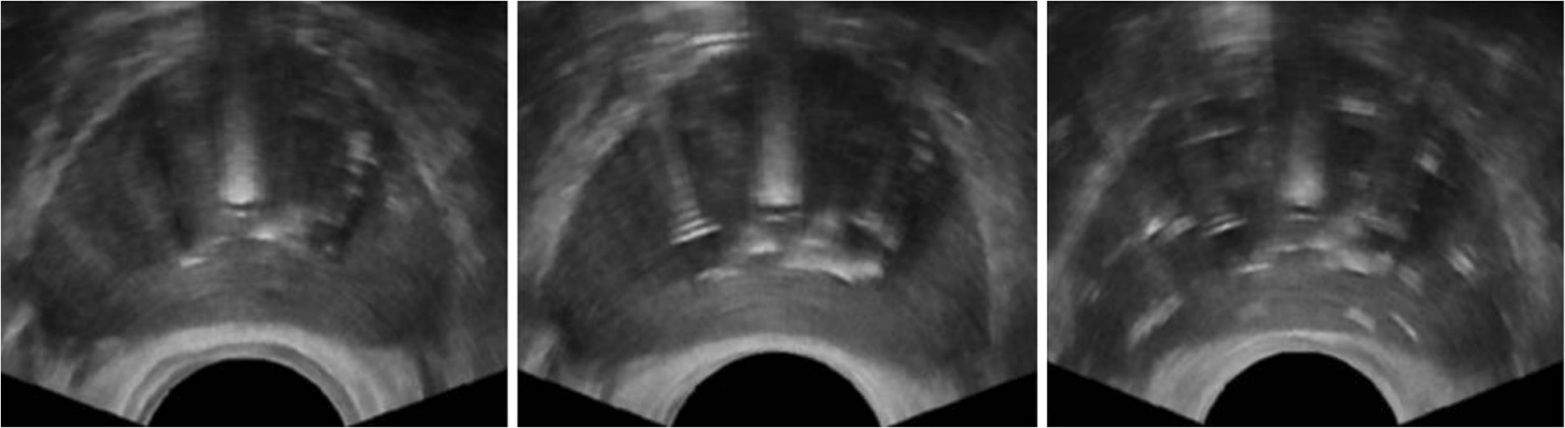
**Example of TRUS (transrectal ultrasound) image acquisition of the prostate (mid-gland) at 3 different time points during the course of the implant (all in the operating room).** Left image corresponds to baseline volume before the implant (V1), middle image to prostate volume after the insertion of 2 fixation needles (V2) and right image to prostate after the completion of the implant (V3).

The implant was realized using three-dimensional (3D) ultrasound-guided (BK Medical Systems, Harlev, Denmark) IO interactive planning with virtual needle guidance, robotic seed delivery and needle retraction system (FIRST, Nucletron, Veenendaal, The Netherlands). An IO 3D reconstructed TRUS was done in extended dorsolithotomy position with the patient under general or spinal anesthesia. The complete implant planning and general guidelines were described in a previous publication [7]. Patients were told to stop anti-inflammatory drugs 10 days before the implant. No perioperative cortisone was given. Before any needles were inserted, a 3D-TRUS of the entire prostate was done and saved. The prostate volume was contoured after the implant, usually within 2 days after the procedure on each 2.5 mm thick slice. This prostate volume was defined as V1. Then another 3D-TRUS was done with 2 fixation needles inserted into the prostate. These needles have a special harpoon to hold the prostate in place. Each implant was done with the same model of fixation needles. The prostate volume was contoured in the operating room and defined as V2. The intra-operative dosimetry planning of the implant was then done with V2. The planned tumor volume consisted of the prostate gland with margins of 3 mm in all directions. Following completion of implant with all seeds implanted, a third 3D-TRUS was done and saved, and the prostate volume was contoured within the next 2 days and defined as V3 (Figure 1). Next, each patient underwent a computed tomography (CT) scan usually at 30 days after the implant (4 to 6 weeks) to evaluate implant quality and dosimetry. The CT scan was performed with 3 mm thick slices. All prostate contouring on TRUS and CT was done by a single physician (DT). Seed localization was performed using Nucletron FIRST integrated software Spot Pro, version3.1. The D30 dosimetry was considered satisfactory if the D90 (minimum dose in Grays received by 90% of the prostate volume) was > 130 Gy. Urinary retention was evaluated as the duration of catheterization following the implant procedure. The duration was grouped into 3 categories: no immediate post-operative catheterization, duration of 1–6 days and more than 7 days (includes self-catheterization).

### Statistical analysis

Descriptive analyses were performed to generate summary statistics. Correlations between clinical and implant variables and differences in prostate volumes were measured by Pearson correlation coefficient. The seed activity variable was dichotomized as low (< 0.5 mCI) and high (> = 0.5 mCi). Univariate and multivariate linear regression analyses were performed to determine significant predictors of change in prostate volume. For the multivariate analyses, all models included age and baseline volume. Because at the time of measuring V1 the number of seeds, needles and seed activity were irrelevant to V1, the models analyzing the difference between V3-V1 and V2-V1 did not include these factors. Because the number of seeds and needles were highly correlated, two models were estimated for the difference in volume between V3 and V2; one included the number of seeds and the other the number of needles.

The minimum dose delivered to 90% of the prostate volume (D90) at day 30 was analyzed as a continuous variable and then as a dichotomous variable (< 130 Gy and >130 Gy). Linear regression and logistic regression analyses were performed to analyze the influence of clinical and implant factors on D90 at day D30. Because the differences in prostate volume between each step were highly correlated, each multivariate analysis was modeled with each difference in prostate volume separately (e.g. difference V2-V1 together with the clinical and implant factors, then difference V3-V1 and other factors and finally V3-V2 and other factors). The limited number of patients with a D90 at day 30 less than 130Gy prevented the fitting of a model with a large number of variables. Therefore, these models only included important variables such as the difference in prostate volume, the baseline volume and the source activity. The proportional odds model (POM) was used to assess the influence of edema on the duration of catheterization. A probability <0.05 was considered to be statistically significant. Statistical program R 2.13.0 was used for all data analysis (http://www.r-project.org).

## Results

### Prostate volume changes and its associated factors

Patient demographics and implant characteristics are summarized in Table  1. Changes in prostate volume occurring throughout the implant (V1 to V3), including insignificant changes (< 5%) can be found in Table 2.

**Table 1:** Patient baseline characteristics

**Characteristic**	**Median (range)**
**Age (years)**	66 (48–78)
**TNM stage**	
T1c	70.5%
T2a	24.2%
T2b	5.4%
**Gleason score**	
≤ 6	88.6%
7	11.4%
**PSA (ng/ml)**	5.56 (0.88 - 18.46)
< 10	89.8%
≥10	10.2%
**IPSS (baseline)**	3 (0–24)
≤ 3	57.6%
>3 and ≤12	38.2%
>12	4.2%
**Prostate baseline volume (cc)**	36.8 (14.4-67.7)
**No. of needles**	23 (16–32)
**No. of seeds**	58 (38–94)
**Activity (mCi)**	0.59 (0.4-0.65)
High (≥0.5mCi)	54.4%
Low (<0.5mCi)	45.6%
**No of seeds/needle**	2.6 (1.9-3.8)

*Abbreviations: TNM* The American Joint Committee on Cancer staging, 7^th^ edition, 2009, *PSA* prostate-specific antigen, *IPSS* International Prostate Symptom Score, *cc* cubic centimeters, *mCi* millicurie.

**Table 2:** Changes in prostate volume

**Difference in percent**				**Difference in cc**			
	**∆ 2-1**	**∆ 3-2**	**∆ 3-1**		**∆ 2-1**	**∆ 3-2**	**∆ 3-1**
Increase*							
**Total**	70.7	85.9	82.1	Mean (SD)	2.51 (5.1)	1.94 (2.55)	4.46 (5.54)
<5%*	20.0	43.6	10.7	Median	2.0	1.66	4.25
>5%*	50.7	42.3	71.4	IQR	−0.32-4.77	0.56-2.99	1.43-7.20
**Mean in cc (SD)**	6.0 (12.5)	4.5 (5.5)	10.3 (12.6)				
Decrease*							
**Total**	29.3	14.1	17.9	Decreasing mean (SD)	−2.49 (2.73)	−1.47 (1.54)	−2.79 (2.65)
<5%*	16.4	10.7	10.8	Increasing mean (SD)	4.56 (4.33)	2.48 (2.25)	6.04 (4.68)
>5%*	12.9	3.4	7.1				

*Increase/decrease in *percentage of patients.*

*Abbreviations*: *cc* cubic centimeters, *∆ 2–1* difference between volumes 2 and 1, *∆ 3-2* difference between volumes 3 and 2, *∆ 3-1* difference between volumes 3 and 1, *SD* standard deviation, *IQR* interquartile range.

Mean baseline prostate volume (V1) was 37.4 ± 9.6 cc. Fifty-one percent of patients had a volume increase of > 5% after the insertion of 2 fixation needles (V2). The mean increase was 6.0% (SD 12.5%).

At the completion of the implant (V3), there was a further increase by an average of 1.94 cc (SD 2.55cc) in Pvol compared to V2. This corresponds to a mean increase of 4.5% (SD 5.5%). The total mean change in Pvol from before the implant before any manipulation (V1) to the end of the implant (V3) was 10.3% (SD 12.6%) (Table 2).

Although the prostate swelling caused by insertion of the fixation needles alone was larger than the one caused by the implant itself, this did not reach statistical significance (p = 0.23).

This might explain why in multivariate linear regression results, baseline Pvol is predictive of Pvol changes between V2 and V1 and V3 and V1 but not between V3 and V2. See Table 3 for univariate and multivariate analysis.

**Table 3:** Univariate and multivariate regression analysis for volume difference

**Modeled with edema between**	**Factor**	**Univariate analysis**		**Multivariate analysis**	
		**Correlation coefficient (95% CI)**	P value*	**Correlation coefficient (95% CI)**	**P value**
**∆ V2 - V1**	Baseline volume	−0.18 (−0.27/-0.10)	**<0.0001**	−0.18 (−0.27/-0.10)	**<0.0001**
	Age	−0.05 (−0.19/0.07)	0.38	−0.005 (−0.13/0.12)	0.94
**∆ V3 - V1**	Baseline volume	−0.16 (−0.25/-0.07)	**<0.001**	−0.16 (−0.26/-0.07)	**0.001**
	Age	−0.05 (−0.19/0.10)	0.51	−0.001 (−0.15/0.14)	0.99
**∆ V3 – V2**	Baseline volume	0.024 (−0.02/0.07)	0.30	0.006 (−0.05/0.06)	0.82
	Age	0.006 (−0.06/0.07)	0.85	−0.002 (−0.07/0.07)	0.94
	Seeds/needles	−0.19 (−1.46/1.09)	0.77	-	-
	Activity	−0.52 (−1.35/0.30)	0.21	−0.11 (−1.3/1.06)	0.84
	No. of seeds	0.03 (−0.002/0.06)	0.07	0.03 (−0.01/0.07)	0.18
	No. of needles	0.12(0.018/0.23)	0.02	0.11 (−0.05/0.28)	0.16

*Abbreviations*: *CI* confidence intervals, *∆ 2–1* difference between volumes 2 and 1, *∆ 3-2* difference between volumes 3 and 2, *∆ 3-1* difference between volumes 3 and 1, *SD* standard deviation, *IQR* interquartile range. * bold when p < 0.05.

### Influence of edema on implant quality parameter D90 on day 30

Clinically, the extent of prostate swelling had an influence on D90. Table 4 presents the complete data.

**Table 4:** Multivariate logistic analysis for D90 at Day 30 as dichotomized variable (model 1: D90 >130 vs. <130 Gy)

	**Factor**		**Multivariate analysis**		
**Model with edema between**		**Odds ratio**	**95% CI**		P value*
**∆ V2 – V1**	Baseline volume	1.02	0.96	1.09	0.51
	Age	-	-	-	-
	Source activity	1.49	0.47	4.92	0.50
	V2-V1	1.17	1.02	1.37	**0.038**
**∆ V3 – V1**	Baseline volume	1.00	0.95	1.06	0.94
	Age	-	-	-	-
	Source activity	1.76	0.57	5.69	0.32
	V3-V1	1.06	0.95	1.20	0.31
**∆ V3 – V2**	Baseline volume	1.00	0.94	1.06	0.99
	Age	*-*	*-*	*-*	*-*
	Source activity	1.61	0.51	5.25	0.41
	Nr of seeds	-	-	-	-
	Nr of needles	-	-	-	-
	V3-V2	0.87	0.72	1.05	0.12

*Abbreviations*: *D90* minimum dose received by 90% of prostate volume at Day 30, *∆ V2-V1* difference between volumes 2 and 1, *∆ V3-V2* difference between volumes 3 and 2, *∆ V3-V1* difference between volumes 3 and 1, *CI* confidence intervals.* bold when p < 0.05.

The median D90 at Day 30 following the brachytherapy was 158 Gy (range 105 to 236 Gy). Univariate logistic regression using D90 as a dichotomized variable (model 1 of D90: <130 vs. >130Gy) revealed that a Pvol change between V1 and V2 (OR 1.17, 95% CI, 1.02-1.37, p = 0.04) were significantly associated with D90. An increase of 10% in prostate volume between V1 and V2 results in an increase of D90 at Day 30 by 11.7%.

When D90 was analyzed as a continuous variable (model 2 for D90), both differences between V1-V2 (p = 0.0002) and V1-V3 (p = 0.001) were strongly associated with D90.

An increase of 10% between V1 and V2 would have an increase of the D90 at Day 30 by 14.6%.

### Relationship between edema and urinary retention

A majority of patients (57.7%) did not require urinary catheterization after the implant (group 1); 33.5% needed 1 to 6 days of catheterization (group 2), and 8.7% required either ≥ 7 days or auto-catheterization (2 weeks - 6 months) (group 3). The baseline prostate volume (V1) was the only predictor of the duration of urinary retention in both univariate (p = 0.04) (not shown in table) and multivariate regression analysis. Therefore, given all other clinical characteristics are the same for 2 patients, the one with 10% larger prostate volume at baseline would be at 48% higher risk of progression from a lower to a higher retention group (Table 5).

**Table 5:** Proportional odds model for urinary retention

**Model with edema between**	**Factor**	**Odds ratio**	**95% CI**		P value*
**∆V2 – V1**	Baseline volume	1.04	0.99	1.07	0.063
	Source activity	0.94	0.48	1.84	0.86
	V2-V1	1.00	0.93	1.07	0.91
**∆V3 – V1**	Baseline volume	1.04	1.00	1.08	**0.032**
	Source activity	0.93	0.48	1.82	0.83
	V3-V1	1.01	0.96	1.08	0.57
**∆V3 – V2**	Baseline volume	1.04	1.00	1.07	0.052
	Source activity	0.98	0.50	1.94	0.96
	V3-V2	1.09	0.96	1.24	0.19

*Abbreviations*: *OR* the odds ratio, *CI* confidence intervals, *V1* baseline volume, *V2* volume after insertion of 2 fixation needles, *V3* volume at the completion of the implant.* bold when p < 0.05.

## Discussion

Little is known about the incidence of prostate edema (PE) during prostate brachytherapy and its risk factors. Although its course following the implant was documented [8,9], little can be done to improve dosimetry after the patient left the operating room. With that in mind, the objective of the present study was to evaluate changes in prostate volume in the operating room before and at the time of the completion of the implant. This study consistently used 3D-TRUS measurements of the prostate to eliminate uncertainties from combining different imaging modalities.

In this present study, we considered changes ≤ 5% in prostate volume as insignificant. This is in accordance with Liu et al. [10] who found the intraobserver variability for TRUS- imaging to be 4.4% for a mean prostate volume of 39.6 cc. In addition, we believe that a small difference in prostate volume would be clinically insignificant.

The most surprising finding in this study was first that prostate volumes increase by > 5% were seen more often after the insertion of fixation needles (50.7%) than after the insertion of all the radioactive sources (42.3%). And second that the mean difference in prostate volume was between V1 and V2 (2.51 cc) and V2 and V3 (1.94 cc), was not significant (p = 0.2).

We believe that the present study is the first to analyze prostate volume changes on three different time-points by the same imaging modality during the course of a permanent seed implant in a relatively large number of patients compared to other similar studies. Martinez et al. [11] in a prospective study of high dose rate (HDR) interstitial brachytherapy in 41 patients, found, similar to our data, that the largest increase in prostate volume occurred shortly after the insertion of needles (before treatment delivery), presumably due to initial mechanical trauma. Interestingly, 28.6% of our patients did not experience >5% increase in prostate volume between V1 and V3.

There are many possible explanations for this finding. For one, there is interobserver variability, which we didn’t measure in this study but has been shown by Liu et al. [10] to be at a mean of 9.3%. Prostate volume can also depend on where to start and end prostate contouring. The starting slice in our study was not set at a specific point. This decrease could be due to a deformation of the prostate that resulted in a falsely measured decrease in Pvol. Volume changes due to the insertion of the fixation needles and the radioactive seeds are small and account for less than 1% of the change in Pvol between V2 and V3.

Applying different pressure with the TRUS-probe at the different measuring points on the prostate can cause indentation that can deform the prostate and cause changes in prostate contouring [10]. Another important point is that the seeds in the prostate can cause artifacts that make prostate volume definition difficult. Post-implant TRUS has been shown to have a higher intraobserver variability compared with MRI, as reviewed by Liu et al. [10]. This could explain why there is so little change in prostate volume between the implant of the 2 fixation needles (V2) and the completion of the implant (V3).

On the other hand, our data are supported by Smith et al. [12] who showed, measured with TRUS, an increase of 18% in prostate volume with the implant of the needles, before seed delivery.

Another explanation for a lack of significant Pvol changes is that there might be individual differences in response of prostate and peri-prostatic tissue to mechanical injury. The present results showed that the extent of Pvol changes is mainly dependent of baseline prostate volume: the smaller the prostate, the larger the edema. This confirms the results from other studies. Chung et al. [13] demonstrated that small prostates (≤ 25 ml) have the greatest post-implant edema compared to larger ones, even though they argued that this might be the result of an overestimation of prostate volume at the time of the implant. Using CT-MRI fusion on the day of implant, on Day 8 and 30, Taussky et al. [14] reported that smaller prostates (<35 ml) show a proportionally greater increase in volume than do larger ones as well.

Edema can have an influence on implant quality and can cause suboptimal implants [14]. Biochemical outcome had been linked to D90 in several published reports of prostate brachytherapy [15,16]. In a study by Zelefsky and collegues, a threshold of ≥130 Gy was used to define a satisfactory implant [17]. Our results showed that an increase of 10% in edema between V1-V2 increases 4.79 times the chance of a D90 to be >130 Gy. This on first sight counterintuitive result may simply be due to the fact that only 16 patients (10.7%) of the study group had a D90 value <130 Gy. On the other hand, we believe this is due to the fact that the prostate volume used for implant planning (V2) overestimates the volume compared to at D30, because it has already experienced a large part of the PE due to the implant of the fixation needles.

We further found that an increase in prostate volume between V1 and V2 by only 10% (less than 1 SD) could increase the risk for longer duration of AUR by 48% on average. Baseline prostate volume, but not the extent of PE, was the only factor associated with the duration of AUR. Unfortunately, data on the dose to the urethra are not available to measure their possible influence on urinary retention. Rates of AUR vary in the literature between 5.6% [18] to 34% [19]. In our data 33.5% of patients required short-term catheterization (1 to 6 days) while only 8.7% needed long-term catheterization (≥ 7 days). No cut-off level could be identified to predict for a larger difference between V1 and V2 and therefore an increase in AUR. We found that the larger the baseline prostate volume the longer duration of catheterization.

This corroborates with findings from prior single-institution experience. Thus, Crook et al. [20] also found that a large prostate volume at the time of implant was independently associated with AUR. Our results cannot be applied generally. Our outpatient unit closes relatively early in the evening (8 PM), giving patients implanted later in the day little time to urinate before they have to be discharged. Taking this into consideration, the percentage of patients needing catheterization >1 day (in over 700 patients implanted so far) is only about 8%, which is comparable to the literature.

Findings of the present study raised several intriguing clinical implications. First, because relatively little trauma to the prostate associated with the insertion of fixation needles causes the majority of PE, we suggest to plan the implant after the insertion of the fixation needles or to add a larger PTV margins if planned without this initial trauma. Although we didn’t compare dosimetry at D90 for prostates planned with or without fixation needles, we believe that fixation needles can help to reduce margins added to compensate for PE and could therefore help to improve implant quality and reduce doses to organs at risk. It could be especially helpful to add larger margins to smaller prostates. This added margin could represent an increase in volume of 10.3% (1 SD in difference between V1 and V3) for larger prostates and for smaller prostates up to 20.6% (2 SD).

Second, the use of anti-inflammatory treatment, especially for small prostates should be further explored. Feigenberg et al. [21] showed that the use of cyclooxygenase-2 (COX-2) inhibitor therapy 1 week before the implant decreased the risk of urinary retention. These findings were also supported by Sacco et al. [22] in a retrospective series of patients using a 2-week course of dexamethasone started after the implant. However, the negative results from a phase III trial of a 4-week course of COX-2 inhibitor therapy (starting either on the day of implant or 1 week prior to implant) did not support this preventive approach. Even if this matter remains inconclusive, none of these studies investigated sub-groups of patients, such as patients with smaller prostates.

We believe that more research into the reduction of PE would not only be beneficial for implant quality but also reduce the duration of AUR.

This study has several limitations that could affect its interpretation. First, this is a retrospective series, thus possibility of bias in patient selection is unknown and cannot be assessed. Second, although this study showed that fixation needle-associated PE is predictive of D90, this study lacks TRUS prostate volume measurements at day 30.

## Conclusions

Smaller prostates have larger PE. The insertion of 2 fixation needles with a harpoon causes at least as much PE than the implant of the radioactive seeds. Therefore, in centers using pre-planning techniques careful consideration should be given to planning volume in order to compensate for prostate volume differences occurring in the operating room. Furthermore, we need to know more about what causes PE and how to prevent it.

## Abbreviations

Pvol: Prostate volume; PB: I^125^ permanent seed brachytherapy; AUR: Acute urinary retention; 3D-TRUS: Three-dimensional transrectal ultrasound; V1: Baseline volume; V2: Prostate volume after the insertion of 2 fixation needles; V3: Prostate volume at completion of the implant; D90: Minimum dose in Grays received by 90% of the prostate volume; IO: Intraoperative; 3D: Three dimensional; CT: Computed tomography; PE: Prostate edema.

## Competing interests

The authors have no conflict of interest. Dr Chira was supported by a fellowship grant from Laboratoires Paladin Inc.

## Authors’ contributions

CC, GD, SL, JFC, DT all drafted the manuscript. DT determined the prostate volumes and provided the clinical data. SL conducted the statistical analysis. All authors read and approved the final manuscript.
